# Anaerobic power estimation using LASSO regression models of lumbar isokinetic parameters at different angular velocities: the role of body composition

**DOI:** 10.7717/peerj.21526

**Published:** 2026-07-21

**Authors:** Oguzhan Arslan, Ahmet Kurtoğlu, Bekir Çar, Ozan Bahadır Türkmen, Jarosław Muracki, Robert Trybulski, Alperen Şanal, Safaa M. Elkholi

**Affiliations:** 1 Ministry of National Education, Ankara, Turkey; 2 Department of Coaching Education, Faculty of Sport Science, Bandirma Onyedi Eylul University, Balikesir, Turkey; 3 Department of Physical Education and Sport Teaching, Faculty of Sport Science, Bandirma Onyedi Eylul University, Balikesir, Turkey; 4 Department of Physiotherapy and Rehabilitation, Faculty of Health Science, Bandirma Onyedi Eylul University, Balıkesir, Turkey; 5 Institute of Physical Culture Sciences, Department of Physical Culture and Health, University of Szczecin, Szczecin, Poland; 6 Medical Department, Wojciech Korfanty Upper Silesian Academy, Katowice, Poland; 7 Institute of Graduate Education, Department of Sport Science, Canakkale Onsekiz Mart University, Çanakkale, Turkey; 8 Department of Rehabilitation Sciences, College of Health and Rehabilitation Sciences, Princess Nourah bint Abdulrahman University, Riyadh, Saudi Arabia

**Keywords:** Anaerobic performance, Body composition, Trunk, Power, Isokinetic parameters, Endurance

## Abstract

**Background and Aim:**

Anaerobic power production is not limited to the lower extremity muscles but is also closely related to the stabilization and endurance capacity of the trunk muscles. In particular, the isokinetic properties of the lumbar region muscles may be associated with short-term high-intensity physical performance. The aim of this study was to evaluate the associations between lumbar region isokinetic strength parameters and body composition variables obtained at angular velocities of 60°/s and 240°/s with anaerobic power.

**Method:**

In the study, regularized regression analysis (LASSO) with leave-one-out cross-validation (LOOCV) was applied using isokinetic variables such as peak torque (PT), total work (TW), and average power (AP) measured at angular velocities of 60°/s and 240°/s, along with body composition parameters (body fat percentage, body mass index, fat-free mass). Anaerobic power was used as the dependent variable.

**Results:**

At 60°/s, isokinetic parameters alone demonstrated limited predictive ability (LOOCV *R*^2^ = 0.19), although several variables such as total work and peak torque were retained in the model. When body composition variables were included, explanatory performance improved substantially (LOOCV *R*^2^ = 0.81), with fat-free mass (FFM) and fat mass emerging as the most stable predictors. At 240°/s, isokinetic parameters alone showed no meaningful predictive ability (LOOCV *R*^2^ = −0.18), indicating poor generalizability. However, when body composition variables were added, model performance increased markedly (LOOCV *R*^2^ = 0.80), with FFM, fat mass, and fat percentage demonstrating consistent associations with anaerobic power.

**Conclusion:**

Lumbar isokinetic strength parameters showed limited and unstable associations with anaerobic power when considered independently. However, the inclusion of body composition variables, particularly fat-free mass, substantially improved model performance. These findings suggest that anaerobic power is more strongly associated with body composition than with isolated lumbar isokinetic strength parameters, and that the contribution of trunk muscle function may be secondary and context-dependent.

## Introduction

Anaerobic power is defined as a fundamental physiological capacity for the successful performance of physical activities that require high-intensity energy production in a short period of time (Støa et al., 2025). Although the focus is usually on lower extremity muscles (Dong et al., 2025), the stabilization and strength production capacity of trunk muscles, especially lumbar region muscles, may be associated with anaerobic performance (Lee et al., 2021). The dynamic and isometric endurance of these muscles supports neuromuscular integrity and power transmission in sports involving sprinting, changing direction, and repeated high-intensity efforts (Fujita et al., 2019). Therefore, anaerobic power relates not only to peripheral muscles but also to central postural support systems (Zemková, 2022).

Isokinetic performance measurements offer an objective and reliable method for evaluating force production in different regions at varying angular velocities (Schindler et al., 2023). These tests calculate values such as peak torque, total work, and average power (Dvir, 2025). Each angular velocity represents distinct biomechanical and physiological conditions (Parrino et al., 2023); for instance, lower velocities reflect maximum force production (Hong, Woo & Jeon, 2024), while higher ones indicate fast-twitch motor unit activation, dynamic force production, and muscle endurance (Andrade et al., 2012). This directly mirrors physiological characteristics of muscle types and the viscoelastic structure of the muscle–tendon unit (Taylor et al., 1990).

Body composition is a key factor in muscle function and anaerobic performance (Maciejczyk et al., 2015). Fat-free mass, primarily skeletal muscle, underpins mechanical potential *via* actin–myosin interactions and phosphagen-based energy production (Stubbs et al., 2018; Tallis, James & Seebacher, 2018). Higher body fat percentage reduces functional efficiency through passive musculoskeletal loading and impairs thermoregulation and oxygen diffusion, negatively affecting anaerobic performance (Knechtle & Nikolaidis, 2018). Although body mass index (BMI) provides a general body composition indicator, it falls short for sport-specific muscle-fat distribution assessments. Isokinetic measurements precisely capture mechanical muscle capacity (De Ste Croix, Deighan & Armstrong, 2003) but neglect underlying metabolic sources, muscle volume, fiber type distribution, and energy efficiency (Baltzopoulos & Brodie, 1989), which may limit their independent explanatory power when used alone in predictive models. For example, individuals with similar torque may differ in sustainment: higher fat-free mass (FFM) enables lower energy expenditure, whereas higher fat demands greater metabolic effort (Kim et al., 2022).

Adding body composition to other variables, such as muscle mass, energy stores, muscle fiber activity, and passive loading, increases the explanatory ability of the regression model and gives a better understanding of the expenses related to generating force. Previous studies have focused mainly on the muscles of the lower extremities for predicting anaerobic performance; there are just three articles examining the trunk muscles’ isokinetic strength (Park et al., 2019; Song, Chun & Lee, 2021; Yoon & Kim, 2021), with no research including lumbar strength and body composition in multiple regressions. It is very important since the muscles of the trunk comprise around 40% of body weight and link up the kinetic chain in high-intensity exercise (Lee et al., 2021).

This study examines whether established anaerobic power predictors—validated for lower extremities—extend to lumbar isokinetics when combined with body composition variables, applying multivariate methods to this underexamined region. In this context, the hypotheses of the study are as follows: (1) Lumbar isokinetic parameters may show associations with anaerobic power, although these associations are expected to be limited when considered independently; (2) body composition variables, particularly fat-free mass, are expected to significantly contribute to the explained variance in the model.

## Materials & Methods

### Study design

This study was conducted using quantitative methods and a correlational survey model. To ensure the transparency of the research data, the study has been registered *via* the Open Science Framework (OSF) and the analysis materials, raw dataset and analysis code are openly available in the Zenodo repository at https://doi.org/10.5281/zenodo.19953132 under a CC-NY licens license. The relationships between participants’ isokinetic lumbar muscle strength, body composition, and anaerobic power levels were tested using regularized regression analysis (Least Absolute Shrinkage and Selection Operator, LASSO) with leave-one-out cross-validation (LOOCV). The study has a cross-sectional design, and measurements were collected at a single time point. Separate models were created by considering both low angular velocity (60°/s) and high angular velocity (240°/s) isokinetic test data in the analyses. Intermediate velocities were excluded due to practical constraints: (1) each additional velocity requires separate test protocols and recovery periods, which would substantially increase testing duration and participant fatigue given our sample size (*n* = 30); and (2) 60°/s and 240°/s represent the most frequently reported velocities in lumbar isokinetic literature, enabling direct comparison with existing studies (Song, Chun & Lee, 2021; Yoon & Kim, 2021). Thus, the effect of different muscle activation characteristics on predicting short-term high-intensity physical performance could be evaluated.

### Participants

This study included a total of 30 healthy male volunteers aged between 20 and 23 years who had a history of regular physical activity and no diagnosis of any musculoskeletal, cardiovascular, or metabolic diseases. The average age of the participants was 21.43 ± 0.77 years, average height was 178.06 ± 5.31 cm, body weight was 71.29 ± 7.24 kg, BMI 22.45 ± 2.11 kg/m^2^, body fat percentage 10.64 ± 3.85, fat mass 7.88 ± 3.40 kg, and fat-free body mass (FFM) 63.41 ± 5.00 kg (Table 1). All participants were recreational-level active individuals who had been engaging in regular exercise at least three days a week for the past year. Inclusion criteria for the study included: regular physical exercise (at least three days per week for the past year), no history of back pain or musculoskeletal disorders, and no health issues that would prevent participation in the test protocols. Conversely, individuals with lumbar disc herniation, a history of lumbar surgery, severe orthopedic injuries, cardiovascular or metabolic diseases, professional athletes (elite-level competitive athletes), and individuals with a BMI value above 30 kg/m^2^ were excluded from the study. These criteria were carefully applied to minimize potential deviations in isokinetic measurements.

**Table 1:** Baseline characteristics of participants.

Parameters	Mean	SD	Minimum	Maximum
Age (year)	21.43	0.77	20	23
Height (cm)	178.06	5.31	166.00	188.00
Weight (kg)	71.29	7.24	57.60	91.50
BMI (kg/m^2^)	22.45	2.11	18.60	26.40
Fat (%)	10.64	3.85	2.70	18.00
Fat Mass (kg)	7.88	3.40	1.60	14.70
FFM (kg)	63.41	5.00	51.40	76.80
PAP (W)	984.17	102.27	795.50	1,232.00
**60°/s velocity**				
PT-EXT	414.91	83.46	249.20	668.00
PT-FLEX	223.65	36.45	155.50	308.30
TW-EXT	2,033.77	308.84	1,478.80	2,804.70
TW-FLEX	1,191.21	197.63	853.90	1,504.20
AP-EXT	232.64	51.06	35.60	333.50
AP-FLEX	143.80	22.08	105.20	174.00
**240°/s velocity**				
PT-EXT	425.89	92.15	275.20	600.80
PT-FLEX	311.81	87.23	98.40	459.20
TW-EXT	3,784.83	1,379.98	1,035.90	6,727.90
TW-FLEX	2,030.55	672.14	654.20	3,218.80
AP-EXT	357.02	137.00	90.20	669.40
AP-FLEX	167.14	59.40	57.20	277.50

**Notes.**

BMI Body Mass Index FFM Fat-Free Mass PAP Peak Anaerobic Power PT-EXT Peak Torque–Extension PT-FLEX Peak Torque–Flexion TW-EXT Total Work–Extension TW-FLEX Total Work–Flexion AP-EXT Average Power–Extension AP-FLEX Average Power–Flexion

*A priori* power analysis was conducted using G*Power (v3.1.9.7) to estimate the minimum required sample size for detecting moderate effect sizes in a conventional multiple linear regression framework (*f*^2^ = 0.30, α = 0.05, 1− β = 0.80), which indicated a minimum of 24 participants. However, given the study’s use of regularized regression methods (LASSO), which apply shrinkage and variable selection to reduce overfitting in small samples, this calculation was considered a general reference rather than a strict requirement. A total of 30 participants were included to enhance model stability and account for potential data exclusion. Given the number of candidate predictors relative to the sample size, regularization-based modeling was preferred. Therefore, LASSO regression with leave-one-out cross-validation (LOOCV) was applied to perform variable selection and model estimation within a unified framework. All predictor variables were standardized prior to model fitting to ensure comparability of coefficient estimates.

The study was conducted in accordance with the 2013 revision of the Helsinki Declaration, and all ethical principles were strictly adhered to. Prior to the study, the necessary ethical approval was obtained from the Non-Interventional Ethics Committee of the Faculty of Health Sciences, Bandırma Onyedi Eylül University (Ethics Committee Decision No: 2025/2, Date: 17.02.2025). Participants were provided with verbal and written information about the purpose of the study, the application process, potential risks, and benefits; written consent forms were obtained to ensure voluntary participation. Participants’ personal data were kept confidential and reported in accordance with anonymization principles. A total of 30 participants were included to enhance model stability and account for potential data exclusion. However, with 14 predictors tested across 30 observations, the ratio of cases to variables remains low. This structural constraint is acknowledged here and further discussed in the limitations.

### Data collections tool

#### Anaerobic power measurement

The Wingate Anaerobic Test (WAnT) protocol was used to assess participants’ anaerobic performance levels. This test is one of the most widely used and well-established methods for assessing anaerobic power during short-term, high-intensity exercise (Zupan et al., 2009). Measurements were performed using a Monark 894E (Monark Exercise AB, Vansbro, Sweden) mechanical brake system bicycle ergometer. Each participant underwent a 5-minute light warm-up protocol prior to the test, which included several short sprints (2–3 s) during the warm-up period.

During the test, resistance equivalent to 7.5% of the participants’ body weight was applied. This load was suddenly applied at the start of the test, and participants were instructed to pedal at the highest possible cadence for 30 s. Power output was derived from the product of braking force and flywheel velocity, and the highest value reached during the 30-s test was recorded as Peak Anaerobic Power (PAP). In this study, only PAP was used as the dependent variable, as it primarily reflects maximal phosphagen system output and peak neuromuscular activation. Although other indices such as mean power and fatigue index provide additional information on anaerobic performance, they were not included in the present models in order to focus on peak power output and to maintain model parsimony. Mean power and fatigue index were excluded as PAP most directly reflects the phosphagen system and maximal neuromuscular activation targeted by lumbar isokinetic assessments, consistent with previous studies. PAP represents the maximum power capacity reached during the first few seconds of the test, when the muscles produce energy based on phosphocreatine, and was calculated in Watts (W).

All tests were conducted in the morning (10:00–13:00), under constant temperature conditions (22 ± 1 °C), in a quiet and controlled laboratory environment. Participants were instructed not to engage in heavy physical activity 24 h prior to the test and to avoid caffeine and stimulant consumption on the day of the test.

Measurements were conducted by an experienced researcher in sports physiology, and verbal support was provided throughout the test to enhance motivation. All participants received the same standardized verbal instructions and encouragement, and the testing procedure was applied in an identical manner to minimize inter-test variability.

The PAP values obtained using this method were used as the dependent variable in regression models, and the associations between isokinetic muscle strength and body composition variables were statistically tested.

#### Isokinetic test measurement

Isokinetic dynamometry was used to evaluate the muscle strength, work, and power output of the participants’ lumbar muscle groups. Measurements were performed using the Humac Norm Cybex CSMi^®^ (Computer Sports Medicine Inc., Stoughton, MA, USA) isokinetic dynamometer device. This device enables the precise and repeatable measurement of biomechanical variables such as torque, work, and power produced by muscles during movement at constant angular velocities (Habets et al., 2018; Roth et al., 2017). Prior to the test procedure, the device was calibrated daily in accordance with the manufacturer’s guidelines, and its accuracy and reliability were verified.

Participants were secured to a specially designed test station in a manner that allowed them to perform maximal voluntary muscle contractions during the test. Extension (backward bending) and flexion (forward bending) movements were measured to evaluate lumbar muscle function. Participants were stabilized in a seated position with safety belts securing the pelvic and thoracic regions. The thighs were fixed to prevent movement of the trunk and pelvis; only trunk flexion-extension movements were permitted. During the test, the arms were crossed over the chest, and verbal instructions ensured that the movement was performed solely from the lumbar region.

Isokinetic tests were performed separately at angular velocities of 60°/s (low speed, high force) and 240°/s (high speed, low resistance). For each speed, participants were allowed to perform three submaximal practice repetitions at the same movement speed before starting the test to familiarize themselves with the movement pattern. Following this, a set of 5 maximal contractions was performed for each test protocol. A 2-minute rest period was provided between sets to ensure full muscle recovery. All tests were conducted in a single session, in the morning, under constant laboratory conditions. Standard motivational feedback was provided to participants throughout the test, and they were encouraged to exert maximal effort for each repetition.

The parameters obtained included Peak Torque (PT, Nm), Total Work (TW, J)—total energy production of the muscle, and Average Power (AP, W)—average power production of the muscle over time. These parameters were analyzed separately for both flexion and extension directions at both angular velocities. All data were obtained through the device’s integrated software, and data integrity was checked before analysis. All isokinetic variables were recorded as PT, TW, and AP, and were treated as candidate predictors in the statistical models rather than direct performance outcomes.

#### Body composition measurements

Participants’ body composition assessments were conducted using the bioelectrical impedance analysis (BIA) method in conjunction with anthropometric measurements. Measurements were taken using the Tanita MC-780 MA P Segmental Body Composition Analyzer^®^ device. This device provides a multi-segmental analysis of body composition using 8-electrode segmental BIA technology and has been reported to provide acceptable validity and reliability for segmental body composition assessment under standardized measurement conditions (Mecherques-Carini et al., 2025). Measurements were taken in accordance with the protocols of the International Society for the Advancement of Kinanthropometry (ISAK) in the morning, in a fasting state (at least 3 h of fasting) and after urination. Participants were instructed to avoid intense physical activity for 12 h prior to the test (Silva & Vieira, 2020). However, BIA-derived estimates are sensitive to hydration status, recent food intake, and acute physical activity, and therefore should be interpreted as field-based estimates rather than criterion-standard measures.

The basic parameters obtained in the assessments were classified as height (cm), body weight (kg), body mass index (BMI, kg/m^2^), fat percentage (%Fat), fat mass (Fat Mass, kg) and fat-free body mass (FFM, kg). BMI was calculated using the classic formula of dividing body weight in kilograms by the square of height in meters. Fat percentage represents the proportion of fat tissue in body weight, while fat-free mass includes skeletal muscle, water, bone, and organ mass. FFM was considered a key explanatory variable in the analyses because of its close association with muscle function and anaerobic performance; however, it may also partly reflect overall body size and musculoskeletal scale.

During the measurements, participants stood barefoot on the weighing platform, ensuring contact between their feet and the electrodes on their hands. Information regarding age, gender, and physical activity level was manually entered into the device system. Each measurement took approximately 30 s, and all measurements were performed by the same technician under constant laboratory conditions (22–24 °C). Participants were also instructed to refrain from caffeine and alcohol intake before testing in order to minimize acute fluctuations in body water distribution. The digital output obtained after the measurement was recorded along with data on the participants’ segmental body regions and general composition data.

Body composition variables were modelled as independent variables in regression analyses alongside the anaerobic performance and isokinetic strength parameters obtained in the study, and their potential associations were examined in multivariate analyses.

### Statistical analyses

The final analytical dataset comprises 30 rows (participants) and 21 columns (variables). The first column contains anonymized participant IDs (ID_001 through ID_030). The remaining columns include one dependent variable (PAP_W), seven body composition variables (Age_yr, Height_cm, Weight_kg, BMI_kgm2, Fat_pct, FatMass_kg, FFM_kg), and twelve isokinetic predictor variables measured at two angular velocities: 60°/s (PT_EXT_60, PT_FLEX_60, TW_EXT_60, TW_FLEX_60, AP_EXT_60, AP_FLEX_60) and 240°/s (PT_EXT_240, PT_FLEX_240, TW_EXT_240, TW_FLEX_240, AP_EXT_240, AP_FLEX_240). All isokinetic variables are expressed in their original units: Peak Torque in Newton-meters (N m), Total Work in Joules (J), and Average Power in Watts (W). Missing data are coded as NA and were absent in the present dataset. The dataset was saved in UTF-8 encoded CSV format with period (.) as the decimal separator to ensure cross-platform compatibility.

A detailed codebook accompanies the dataset, specifying: (i) variable names and descriptive labels; (ii) measurement units and scales; (iii) data collection instruments and software versions (RStudio v2023, Humac Norm Cybex CSMi^®^ software, Tanita MC-780 software); (iv) the exact test order and temporal sequence of measurements; and (v) the inclusion and exclusion criteria applied during recruitment. The repository also includes the complete R analysis script (analysis_script. R), which loads the dataset, applies z-score standardization, executes LASSO regression with LOOCV, and generates all reported figures and tables.

The statistical analyses of the data obtained in the study were performed in the R Studio (version 2023.06.1+524; RStudio, Inc., 2023) environment. The significance level was set at *p* < 0.05 for all analyses. Before starting the data analysis, missing data checks and outlier analyses were performed to ensure data integrity. The normality assumption of continuous variables was evaluated using the Kolmogorov–Smirnov and Shapiro–Wilk tests; the distribution was considered normal if the skewness and kurtosis coefficients were within the ±2.0 limits (George & Mallery, 2024). Accordingly, parametric statistical methods were applied.

In this study, anaerobic power outputs (peak anaerobic power in watts) were used as the dependent variable. The independent variables were the muscle function parameters (PT, TW, AP) obtained from isokinetic tests at angular velocities of 60°/s and 240°/s, and body composition variables (% body fat, FFM, BMI). This study employed a cross-sectional design; therefore, all observed relationships are correlational in nature, and causal inferences cannot be drawn from these data.

The analytical approach of the present study was exploratory and data-driven, aiming to identify the most relevant predictors of anaerobic power. Given the relatively small sample size and the number of candidate predictors, regularization-based modeling was preferred. Therefore, Least Absolute Shrinkage and Selection Operator (LASSO) regression was applied for variable selection and model estimation.

Given that the amount of available predictors exceeded the number of subjects in the experiment, regularization-based modeling techniques were employed. Thus, LASSO regression analysis was performed to select and estimate the most relevant set of predictors. LASSO regression was fitted using the glmnet package (α = 1.0), with all predictors standardized to z-scores. The optimal regularization parameter (*λ*) was determined by leave-one-out cross-validation (LOOCV) based on the minimum mean cross-validated error (*λ*.min). Model stability was assessed *via* 1,000 bootstrap resamples; predictors retained in ≥ 50% of iterations were reported as stably selected. Two models (60°/s and 240°/s) were compared using cross-validated root mean square error (RMSE), mean absolute error (MAE), and R-squared (R^2^).

Standardization of all predictors was performed before estimating the models. Leave-One-Out Cross-Validation (LOOCV) technique was used to determine the optimum value of regularization parameter *λ*. In LOOCV procedure, one person was removed from the dataset in each step. The model was refit on the remaining persons and anaerobic power was predicted for the removed person. The predictive performance of models was characterized by RMSE, MAE, and R^2^ of the LOOCV.

Two separate models for two angular velocities (60°/s and 240°/s) were developed. Moreover, additional models that also included the body composition predictors were fitted in order to assess how much they contribute to predicting anaerobic power. Stability of the model predictions was evaluated through bootstrap sampling, and the percentage of times each predictor was retained was shown.

Model behavior in terms of the distribution of residuals was analyzed visually using residual plots and Q-Q graphs. Model coefficients were visualized with the help of the ggplot2 package. Models that corresponded to two angular velocities were compared, and the influence of body composition predictors was discussed in detail in terms of LOOCV metrics.

To sum up, associations between predictors and anaerobic power were studied in multivariate modeling techniques with the help of regularization methods.

## Results

**Figure 1:**
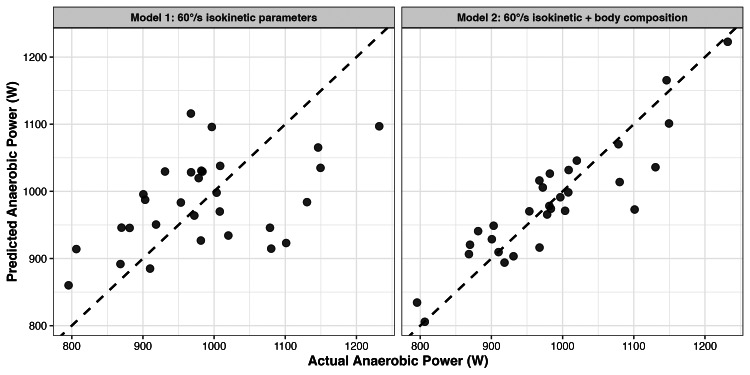
LASSO regression coefficients for predictors of anaerobic power at 60°/s angular velocity. (A) Model 1: Isokinetic parameters only, (B) Model 2: Isokinetic + body composition variables.

The results of LASSO regression analysis are presented in Table 2. For this model, the association between anaerobic power and lumbar isokinetic parameters assessed at 60°/s angular velocity was considered. Initially, the model involved six candidate variables: peak torque (PT), total work (TW), and average power (AP). After the selection of independent variables, several parameters were retained within the model; however, total work-extension (TW-EXT) (coefficient = 0.117) and peak torque-flexion (PT-FLEX) (coefficient = 0.457) were identified as the strongest contributing variables relative to other predictors (Fig. 1A). Overall, however, the predictive performance of the proposed model remained quite low, with an LOOCV R^2^ value of 0.191 and relatively high RMSE (90.42) and MAE (77.34) values, suggesting weak generalizability under strict validation conditions. According to bootstrap-based stability analysis, TW-EXT proved the highest selection frequency among the variables (83.5%), followed by PT-FLEX (69.6%) and TW-FLEX (64.4%), which suggests that TW is the key variable in the isokinetic domain, especially during extension exercises. Overall, however, due to low predictive accuracy, isokinetic parameters are insufficient for explaining anaerobic power at 60°/s.

**Table 2:** LASSO regression models predicting anaerobic power from 60°/s lumbar isokinetic and body composition parameters.

**Predictor**	**LASSO coefficient**	**Selected**	**Stability (%)**
**Model 1**	**LOOCV R^2^ = 0.191**	**RMSE = 90.42**	**MAE = 77.34**
PT-EXT	0.105	Yes	62.1
PT-FLEX	0.457	Yes	69.6
TW-EXT	0.117	Yes	83.5
TW-FLEX	0.083	Yes	64.4
AP-EXT	0.000	No	38.7
AP-FLEX	0.209	Yes	52.7
**Model 2**	**LOOCV R^2^ = 0.812**	**RMSE = 43.59**	**MAE = 33.52**
PT-EXT	0.000	No	55.2
PT-FLEX	0.000	No	42.8
TW-EXT	0.000	No	47.3
TW-FLEX	0.029	Yes	82.5
AP-EXT	0.000	No	37.4
AP-FLEX	0.000	No	32.1
Fat (%)	0.000	No	57.2
Fat mass (kg)	8.981	Yes	81.8
BMI (kg/m^2^)	0.000	No	42.9
Fat-free mass (kg)	12.937	Yes	100.0

**Notes.**

Model 1, 60°/s isokinetic parameters; Model 2, 60°/s isokinetic + body composition; PT-EXT, Peak Torque during extension; PT-FLEX, Peak Torque during flexion; TW-EXT, Total Work during extension; TW-FLEX, Total Work during flexion; AP-EXT, Average Power during extension; AP-FLEX, Average Power during flexion; FFM, Fat-Free Mass; BMI, Body Mass Index.

Finally, body composition-related variables were introduced into the model (Table 2). Specifically, the new independent variables included fat-free mass (FFM) (coefficient = 12.94) and fat mass (coefficient = 8.98), with only one lumbar isokinetic parameter (total work-flexion, coefficient = 0.029) retained within the framework of the extended model (Fig. 1B). The introduction of body composition variables resulted in significant improvement of the model performance, as evidenced by the LOOCV R^2^ value of 0.812 and decreased RMSE (43.59) and MAE (33.52) values. Bootstrapping also provided additional support for the obtained results, demonstrating that 100% and 81.8% of resampled models contained FFM and fat mass, respectively, whereas selection frequencies of most isokinetic variables remained low or moderate. Altogether, these results suggest that body composition-related parameters, primarily FFM, play the dominant role in explaining anaerobic power, with contributions of lumbar isokinetic parameters being of secondary importance.

**Figure 2:**
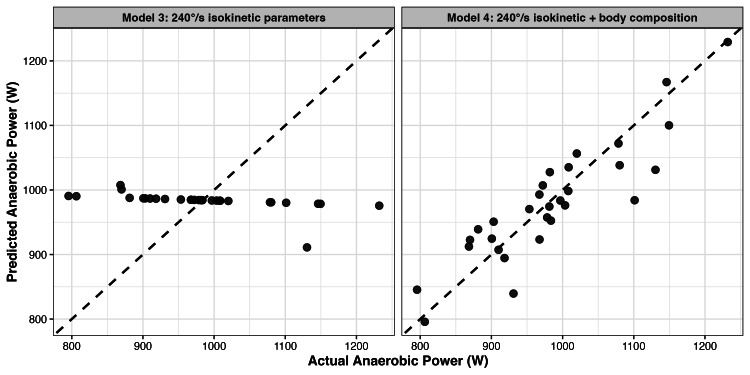
LASSO regression coefficients for predictors of anaerobic power at 240°/s angular velocity. (A) Model 3: Isokinetic parameters only; (B) Model 4: Isokinetic + body composition variables.

Results from the analysis carried out by the LASSO regression model developed for examining the effect of lumbar isokinetic muscle strength parameters at the angular velocity of 240°/s on anaerobic power are summarized in Table 3. In the first step of model development, six predictors of anaerobic power were examined, including peak torque (PT), total work (TW), and average power (AP). After applying LASSO variable selection, no isokinetic predictor remained in the final model (all beta-coefficients equal zero), which means that none of them can explain anaerobic power (Fig. 2A). Furthermore, the performance of the model was poor since its LOOCV R^2^ was negative (−0.180), RMSE = 109.25, and MAE = 82.29. It is noteworthy that these values show that the model performed even poorer than the naïve one based on mean value prediction.

**Table 3:** LASSO regression models predicting anaerobic power at 240°/s angular velocity.

**Predictor**	**LASSO coefficient**	**Selected**	**Stability (%)**
**Model 3**	**LOOCV R^2^= −0.180**	**RMSE = 109.25**	**MAE = 82.29**
PT-EXT	0.000	No	63.4
PT-FLEX	0.000	No	45.2
TW-EXT	0.000	No	31.8
TW-FLEX	0.000	No	30.6
AP-EXT	0.000	No	50.3
AP-FLEX	0.000	No	41.0
**Model 4**	**LOOCV R^2^= −0.797**	**RMSE = 45.25**	**MAE = 36.08**
BMI (kg/m^2^)	0.000	No	58.5
Fat Mass (kg)	8.186	Yes	81.8
Fat (%)	1.618	Yes	78.0
FFM (kg)	14.617	Yes	100.0
AP-EXT	0.000	No	45.1
AP-FLEX	0.009	Yes	71.5
PT-EXT	−0.080	Yes	75.3
PT-FLEX	0.000	No	69.2
TW-EXT	0.000	No	45.9
TW-FLEX	0.000	No	34.1

**Notes.**

Model 3, 240°/s isokinetic parameters; Model 4, 240°/s isokinetic + body composition; PT-EXT, Peak Torque during extension; PT-FLEX, Peak Torque during flexion; TW-EXT, Total Work during extension; TW-FLEX, Total Work during flexion; AP-EXT, Average Power during extension; AP-FLEX, Average Power during flexion; FFM, Fat-Free Mass; BMI, Body Mass Index.

Furthermore, the model stability was analyzed using bootstrap re-sampling approach. According to the obtained results, none of the variables were selected in a stable manner. Thus, peak torque-extension (PT-EXT) was selected in 63.4% of all bootstrap sample, while the rest of the predictors were chosen even less frequently (less than 50% of bootstrap samples). Therefore, the proposed model seems to be unstable and highly sensitive to sampling variations.

## Discussion

The present study explores the associative framework between lumbar isokinetic parameters, body composition, and anaerobic power. While core relationships between muscle function and performance are well-established in lower-limb models (Michalik et al., 2023), this investigation refines the methodology by operationalizing the lumbar region’s specific contribution through a multi-speed, multivariate approach. Previous studies established bivariate correlations between trunk strength and anaerobic power (Song, Chun & Lee, 2021); this study extends that literature by employing multivariate modelling to explore the relative contribution of lumbar parameters in the presence of body composition variables, examining speed-specific relationships that may reflect distinct physiological demands, and demonstrating the dominant role of FFM alongside more limited and less stable contributions of isokinetic parameters—a pattern that has not been consistently examined in previous lumbar-focused studies. These findings highlight that lumbar musculature may contribute to anaerobic performance in a more context-dependent and indirect manner compared to lower-limb-focused models.

While previous studies, including Song, Chun & Lee (2021), who reported significant bivariate correlations (*e.g.*, *r* = 0.629 between trunk extension strength and WAnT peak power, *r* = 0.507 between trunk flexion strength and WAnT peak power) in college male soccer players players, Park et al. (2019), who linked isokinetic trunk strength profiles to pedaling power in elite cyclists, Yoon & Kim (2021), who identified associations between trunk extension/flexion strength and WAnT peak/mean power in snowboarders, as well as cross-sectional analyses reviewed in the systematic literature (Gao et al., 2025) on instability core training effects on trunk strength and sprint performance, primarily established bivariate correlations between trunk/core strength and anaerobic or sprint performance, the present study extends this literature by applying a regularized multivariate modelling approach to lumbar isokinetic parameters and body composition variables. Specifically, this study examined whether lumbar isokinetic variables measured at different angular velocities retain explanatory value when body composition is considered within the same model. Rather than demonstrating an independent or causal contribution of lumbar strength, the present findings suggest that lumbar isokinetic variables show limited and context-dependent associations with anaerobic power, whereas body composition variables, particularly FFM, provide more stable explanatory information. These findings indicate that lumbar musculature may contribute to anaerobic performance indirectly through stabilization and kinetic chain support, but its isolated predictive value appears weaker than that of body composition.

The main objective of this study was to evaluate isokinetic test parameters and body composition variables using a multivariate modelling approach in order to identify alternative biomechanical indicators that are associated with high-intensity exercise performance. The findings partially supported the first hypothesis. Lumbar isokinetic parameters measured at 60°/s showed some contribution in the LASSO model; however, their predictive performance was limited when considered alone. At 240°/s, isokinetic variables alone did not provide meaningful explanatory value for anaerobic power. In contrast, the second hypothesis was more strongly supported. When body composition variables were included, model performance improved substantially at both angular velocities, and FFM emerged as the most stable predictor. Fat mass and fat percentage also contributed to the models, suggesting that anaerobic power is associated not only with mechanical muscle outputs but also with morphological characteristics. Therefore, the present findings refine rather than simply confirm previous bivariate findings by showing that the apparent relationship between lumbar isokinetic function and anaerobic power becomes weaker when body composition is accounted for. Overall, these results suggest that body composition, especially FFM, has a dominant role in explaining anaerobic power, whereas lumbar isokinetic parameters appear to have a secondary and less stable contribution.

The use of isokinetic measures, especially when collected through trunk extension and trunk flexion tests, indicates not only the physical capacity of the muscles in terms of mechanical structure using PT, TW, and AP but also the physiological attributes in relation to motor unit synchronization, fiber type, and energy production. In one study, Song, Chun & Lee (2021) found high correlations between trunk extension PT and WAnT Peak Power (*r* = 0.629, *p* < 0.001) and between trunk flexion power and WAnT Average Power (*r* = 0.720, *p* < 0.001). However, despite this evidence, the current multivariate analysis indicates that these correlations weaken when analyzed together with body composition variables.

According to the power-speed relationship model proposed by Dvir (2025), at low speeds (60°/s), PT/TW parameters are considered indicators of maximal muscle strength, whereas at high speeds (240°/s), parameters such as AP/TW reflect energy transfer capacity associated with fast-twitch muscle fibre activation (Dvir, 2025). In the present study, however, isokinetic parameters measured at 240°/s did not demonstrate stable or meaningful explanatory power in the absence of body composition variables, indicating that theoretical expectations may not fully translate into predictive models within small-sample multivariate contexts. Park et al. (2019) demonstrated a significant relationship between trunk strength and pedaling power in racing cyclist candidates, and Yoon & Kim (2021) reported associations between trunk strength and WAnT-derived parameters in snowboarders. Consistent with these studies, our findings suggest a general association between trunk function and anaerobic performance; however, this relationship appears to be weaker and more model-dependent when examined within a multivariate framework. These associations may reflect underlying mechanisms such as muscle fiber distribution and neuromuscular coordination. However, given the cross-sectional design, we cannot establish whether these mechanisms causally link lumbar function to anaerobic performance. Longitudinal and experimental studies are needed to test these mechanistic hypotheses. In this context, the application of isokinetic PT/TW/AP parameters at different angular velocities provides a distinct assessment framework compared to lower limb evaluations: while lower extremity tests primarily predict propulsive capacity, lumbar assessments capture stabilization efficiency and kinetic chain integrity that may complement lower limb metrics in comprehensive performance analysis (Gao et al., 2025).

Body composition is a fundamental physiological variable associated with anaerobic performance parameters. In particular, in high-intensity, short-duration exercise protocols such as WAnT, an individual’s FFM shows associations with anaerobic power production capacity (Lilić et al., 2020). Since muscle tissue is the primary component of rapid energy production via the phosphocreatine system and glycolytic pathway, numerous studies have demonstrated that individuals with higher FFM exhibit advantages in both PP and AP values (Bar-Or, 1987; Sahlin, 2014). On the other hand, high body fat percentage, which is considered passive mass not involved in energy production, is associated with lower movement efficiency and negatively related to the power-to-weight ratio (Nikolaidis, 2013; França et al., 2024). This situation suggests that normalized values relative to body weight may be more meaningful than absolute values of total anaerobic power production, especially in tests like WAnT.

Actually, in the context of our study, FFM turned out to be the most stable predictor for models with body composition-related factors included, implying that, besides power output capacity, body composition might be linked to the anaerobic performance. At the same time, inclusion of body composition parameters lowered the effect of isokinetic measures on the models in question, thus suggesting that some of the previously observed associations may have been confounded by, or reflected, underlying structural features rather than independent lumbar muscle function. While body composition parameters were relevant for all models regardless of the angular velocity used, different patterns in models’ behavior were found, which may somewhat contradict previous research dedicated to lower limb muscles (Maciejczyk et al., 2015). The latter does not mean, however, that there is any physiological velocity-dependent mechanism behind those differences, as they might just reflect specificities in model behavior and individual sample characteristics. Thus, the present finding suggest that in addition to local muscle function, one should also pay attention to systemic structural factors, namely FFM, in relation to power production mechanisms. Moreover, taking into account the criteria adopted during the process of isokinetic assessment (TW, PT, AP), one might also suggest that these tests are highly dependent not only on muscle performance capability but also on muscle volume and component structure, meaning that the interpretation of results obtained should include FFM measurements as well (Cataldi et al., 2023; Schindler et al., 2023).

The regression approach used in this study allows for the identification of meaningful relationships by simplifying the interaction between variables in biological systems where numerous potential variables, such as isokinetic performance and body composition, are evaluated together (Kipp & Warmenhoven, 2025). In the literature, standard multiple regression models or correlation analyses are often preferred in similar studies (Limberg, Gnilka & Broda, 2021; Sun et al., 2023), but these approaches increase the risk of multicollinearity and complicate interpretation by including all variables in the model at the same time (Bayman & Dexter, 2021; Kyriazos & Poga, 2023). In contrast, the present study employed LASSO, which improves model stability by shrinking less informative coefficients and reducing overfitting, particularly in small samples with multiple predictors (Qian et al., 2024). This approach enables a more parsimonious representation of the data by retaining only the most relevant predictors. Sattler et al. (2016) examined the relationship between isokinetic knee strength and vertical jump performance using regression models, highlighting the importance of appropriate modelling strategies in performance research. Consistent with these recommendations, cross-validation procedures were applied in the present study to evaluate model generalizability and reduce optimism bias (Sattler et al., 2016). Additionally, when isokinetic parameters and body composition variables are analysed together, this approach provides a more robust and interpretable framework for identifying key contributors, offering descriptive information for training planning and performance monitoring. In this regard, the present study applies a regularized multivariate modelling approach to an underexamined anatomical context, providing a complementary perspective to traditional correlation-based analyses.

There are a few limitations to this study. First, concerning interpretative limitations, as the data has been collected by the means of cross-sectional research, no causal interpretation can be made. All correlations observed in the process may indicate reverse causality and presence of some unknown factors that influence the results and make them biased. Such factors may include prior training, genetic predispositions or nutrition. Hence, the correlation coefficients obtained should be regarded as associative but not causal. Second, concerning statistical limitations, although LASSO regularization technique with subsequent cross-validation was used to ensure better model stability, the relatively small size of the sample (*n* = 30 subjects) still makes it vulnerable to overfitting and may lead to unstable coefficient estimates. As a result, the generalization capability of the models is quite limited. Third, concerning measurement limitations, anaerobic performance assessment has been performed with regard only to PAP calculated during the Wingate test. No other anaerobic performance indicators (mean power and fatigue index) have been considered. Consequently, the present findings are specific to short-term maximal phosphagen system output and cannot be generalized to broader anaerobic capacity dimensions such as mean power or fatigue resistance. Moreover, the isokinetic tests conducted during the experiment allowed evaluating dynamometry at two angular velocities only –60°/s and 240°/s. Also, as mentioned above, the isokinetic dynamometer tests for concentric muscle contractions exclusively. The body composition estimation procedure has been based on bioelectrical impedance (BIA). This method is influenced significantly by hydration levels and may provide inaccurate estimations due to its susceptibility to environmental factors. Thus, obtained data may not be regarded as criterion standards, but rather as field-based estimation. Because FFM emerged as the most stable predictor, BIA-derived measurement error –previously reported to reach ±2–3 kg relative to DXA –may have attenuated the true effect size or inflated standard errors in our modest sample, suggesting that the predictive utility of body composition should be interpreted with caution. Finally, as for generalizability limitations, it should be mentioned that the sample involved only physically active and healthy young males. While it is convenient for research purposes, it cannot be generalized to females or other age categories. Also, potential confounding factors such as training level, sports experience, and aerobic power were not included in the study. Such an exclusion of potential confounders from the analysis might contribute to residual confounding and partially explain the variance in anaerobic performance unexplained by the existing models. For example, individuals with systematic resistance training may exhibit superior lumbar neuromuscular control and higher PAP independently, which could inflate the apparent association between lumbar isokinetic strength and anaerobic power in the absence of training status adjustment. Therefore, future studies should recruit larger and more diverse samples (*e.g.*, females, different age groups, and athletes from various sport disciplines), measure additional control variables such as training status, sport type, and aerobic capacity (VO_2_max), and assess complementary anaerobic indices (mean power, fatigue index) as well as intermediate isokinetic velocities and eccentric muscle actions in order to improve external validity and enable causal inference through longitudinal or experimental designs.

## Conclusions

In this study, the associations between lumbar isokinetic force parameters and body composition variables and anaerobic power were investigated at angular velocities of 60°/s and 240°/s. The findings indicate that lumbar isokinetic parameters show limited and model-dependent associations with anaerobic power, whereas body composition variables, particularly FFM, emerge as the most consistent predictors across models. While at 60°/s, isokinetic measures were significant predictors of model performance, the addition of body composition significantly reduced their explanatory effect on the model’s explanatory power. Isokinetic measures, when analyzed alone at 240°/s, did not significantly explain the variance while the addition of body composition variables was able to dramatically improve model performance. This result seems to imply that while there appears to be a relationship between isokinetic performance and anaerobic performance, this relationship is secondary and dependent on physical attributes. The increase in explanatory power due to the addition of body composition measures strongly suggests that FFM is crucially important in determining anaerobic power. In simpler models, the variance attributable to anaerobic power from isokinetic performance might simply reflect the muscle mass characteristic of the individual. Thus, in relation to anaerobic power, lumbar isokinetic performance seems to serve mainly as a supporting attribute which depends on other factors. Clearly, body composition, especially FFM, serves as an essential predictor of anaerobic performance. However, further experimental and longitudinal studies will need to be carried out to confirm that improvements in body composition and trunk function are causally related to improvement in anaerobic performance. Also, future studies will benefit from using large, well-diversified participant pools, testing additional angular velocities (for example 90°/s and 180°/s), using neuromuscular parameters other than those measured herein.

### Practical applications

Based on the results obtained, it may be concluded that the analysis of trunk muscles’ isokinetic function along with the assessment of body composition characteristics, especially FFM, might contribute to the context when analyzing the anaerobic power. Nevertheless, the data from body composition characteristics might be considered a better choice compared to the data from lumbar isokinetics alone. Training programs might benefit by including exercises for developing trunk stability and strength as auxiliary exercises, but lean body mass and body composition should be monitored and developed as well. Still, considering the nature of the study and the fact that the contribution of isokinetic characteristics in the multivariate models is rather weak, one should be careful when applying the conclusions directly to performance prediction purposes.

## Supplemental Information

10.7717/peerj.21526/supp-1 Supplemental Information 1 Raw data.

10.7717/peerj.21526/supp-2 Supplemental Information 2 STROBE Documentation.

## Additional Information and Declarations

### Data Availability

The following information was supplied regarding data availability: The data is available in the Supplemental Files. The data is available at Zenodo: Kurtoglu, A. (2026). Isokinetic Dataset [Data set]. Zenodo. https://doi.org/10.5281/zenodo.19953132
